# A Self-Collision Detection Algorithm of a Dual-Manipulator System Based on GJK and Deep Learning

**DOI:** 10.3390/s23010523

**Published:** 2023-01-03

**Authors:** Di Wu, Zhi Yu, Alimasi Adili, Fanchen Zhao

**Affiliations:** 1 School of Computer Science and Technology, Dalian University of Technology, Dalian 116024, China; 2 Chengdu Research Institute, Dalian University of Technology, Chengdu 611900, China

**Keywords:** self-collision detection, dual-manipulator system, artificial intelligence, deep neural network, GJK algorithm

## Abstract

Self-collision detection is fundamental to the safe operation of multi-manipulator systems, especially when cooperating in highly dynamic working environments. Existing methods still face the problem that detection efficiency and accuracy cannot be achieved at the same time. In this paper, we introduce artificial intelligence technology into the control system. Based on the Gilbert-Johnson-Keerthi (GJK) algorithm, we generated a dataset and trained a deep neural network (DLNet) to improve the detection efficiency. By combining DLNet and the GJK algorithm, we propose a two-level self-collision detection algorithm (DLGJK algorithm) to solve real-time self-collision detection problems in a dual-manipulator system with fast-continuous and high-precision properties. First, the proposed algorithm uses DLNet to determine whether the current working state of the system has a risk of self-collision; since most of the working states in a system workspace do not have a self-collision risk, DLNet can effectively reduce the number of unnecessary detections and improve the detection efficiency. Then, for the working states with a risk of self-collision, we modeled precise colliders and applied the GJK algorithm for fine self-collision detection, which achieved detection accuracy. The experimental results showed that compared to that with the global use of the GJK algorithm for self-collision detection, the DLGJK algorithm can reduce the time expectation of a single detection in a system workspace by 97.7%. In the path planning of the manipulators, it could effectively reduce the number of unnecessary detections, improve the detection efficiency, and reduce system overhead. The proposed algorithm also has good scalability for a multi-manipulator system that can be split into dual-manipulator systems.

## 1. Introduction

Robots, especially manipulators, now play a significant part in medical [1,2], aerospace [3,4], industrial production [5,6], and other industries as a result of the ongoing advancements in science and technology, helping people solve problems by delivering distinct advantages. In recent years, with a deepening of the application of manipulators in various fields, the complexity of tasks has gradually increased, and many tasks require the cooperative operation of dual or multiple manipulators, such as the extraction and transportation of heavy objects [7]. In this context, research on the collaboration of multiple manipulators is of great significance, and the collision detection of manipulators is an indispensable part [8]. The working environment of a multi-manipulator system is typically more complex than that of a single manipulator, except for the collision detection between a manipulator and obstacle, and the self-collision problem caused by the overlapping workspace of manipulators should also be considered.

The purpose of collision detection is to find a collision that may occur during manipulation tasks and avoid collision in the subsequent path planning [9]. At present, the mainstream collision-detection methods of manipulators can be categorized into two types: physical sensor-based and geometric simulation-based [10]. Sensor-based methods utilize physical sensors, which are usually implanted directly inside or outside the robot manipulators, and these methods are generally applicable to dynamic workspaces with external obstacles or human-machine interactions. External cameras are involved sometimes, as authors in [11] placed a dual-depth vision camera to detect the contact position when the manipulator collides with external obstacles. In addition, a torque sensor is also applicable for collision detection. In ref. [12], collision was detected by comparing the deviation between the calculated torque of kinematics and the measured torque of actual joints. The authors of [13] studied the feasibility of collision detection by using the change in the joint motor current value before and after collision, without using an additional physical joint sensor. In paper [14], external acceleration sensors were used to monitor the vibration of the manipulator in real-time, detect the collision of the manipulator through the abnormal vibration frequency, and determine the position and direction of the collision. Although physical sensor-based methods have clear advantages in many scenarios, these methods are costly and cannot be used in simulation studies. Collision detection in simulation scenes uses more geometric simulation-based methods.

The geometric simulation-based methods employ various shapes of the bounding box to envelope the manipulator and obstacle in the simulation environment and calculate the spatial position relationship between bounding boxes with respective algorithms to determine whether collision occurs between colliders [15,16]. The geometry-based method is applicable to situations where coordinates of each manipulator joint and obstacles in Cartesian space are known, such as manipulator path planning. For example, in [17], a manipulator collision constraint for subsequent path planning was established by using the geometric simulation method. Almost all geometric simulation-based methods can be divided into two steps: the establishment of the colliders and the detection of the collision relationship of colliders. The selection of the collider shape directly affects the accuracy of collision detection and the difficulty of the algorithm. Since the geometric simulation method requires real-time continuous modeling for fast-continuous collision detection, it has high requirements for the computing power of control system. Most current collision detection in simulation environments uses a regular-shaped bounding box to envelop the manipulator as the colliders. The authors in [18] used a sphere bounding box envelope manipulator for collision detection in path planning, while those of [19,20] used Oriented Bounding Box (OBB) in their studies. Since collision-detection algorithms are simpler for regular-shaped colliders, these regular-shaped bounding boxes can optimize the modeling and computing speed by simplifying the collider structure and improve the detection efficiency at the cost of a loss of the detection accuracy. This also leads to the problem that all existing methods using regular-shaped colliders have insufficient detection accuracy in manipulator systems with irregular surfaces.

Considering that there is a demand for high precision self-collision detection, in this paper, we used irregular shapes when modeling colliders. Therefore, we used the fine collision-detection algorithm to perform self-collision detection, and the Gilbert-Johnson-Keerthi algorithm (GJK algorithm) [21] is one of those algorithms. The GJK algorithm is an algorithm proposed and continuously improved by Gilbert, Johnson, and Keerthi to quickly detect the distance between two convex polyhedrons [22]. It can output the Euclidean distance of two convex polyhedrons after a finite number of iterations and determine whether a collision occurs from the overlap perspective [23]. Since its introduction, the GJK algorithm has been widely used in various collision detection scenes due to its universality and high accuracy. The authors of [24] proposed a contact-detection and resolution framework based on the GJK algorithm in the Discrete Element Method (DEM), which improves computational compatibility. Meanwhile, those of [25] proposed a GJK-TD method to solve the problem of precision instability that may exist in the application of the GJK algorithm in the DEM. The authors of [26] proposed a method to calculate mesh porosity (volume and area) based on the GJK algorithm for fluid flow modeling. Those of [27] applied the GJK algorithm to the field of robotics to optimize the gripping force of the robotic arm on the target object, via the accurate calculation of the distance between convex objects. The authors of [28] used the GJK algorithm for collision detection in Unmanned Aerial Vehicle (UAV) swarm trajectory planning and improved the distance operator by combining the usage scenarios. After simulation and actual robot validation, the GJK algorithm of the original authors was selected for self-collision detection in our research. However, the fine-detection algorithms represented by the GJK algorithm all face a problem. With an increase in the number of convex polyhedrons and vertices, those algorithms require more time to complete the detection, which cannot meet the requirement of a real-time control system.

Along with artificial intelligence (AI) technologies, most recent delegates, such as deep learning and deep reinforcement learning, are widely deployed in robotics. Benefiting from the excellent feature-extraction capability of deep learning, collision problems at hand have a new solution. Many studies combined neural networks with sensor-based methods. The authors in [29] proposed a deep neural network to learn the collision signal in a torque sensor dataset and extract the collision features of the torque signal, which eventually resulted in high detection performance and real-time generalization capability. The article [30] presented an algorithm based on convolutional neural network and momentum observers, to learn the characteristics of joint motor current values when a collision occurs in a manipulator, saving torque sensors while achieving good detection results for various hard and soft collisions. The authors of [31] used joint-position sensors and deep neural networks to detect collisions by learning the offset signals of the joint positions before and after the manipulator collision. The powerful decision-learning capability of deep reinforcement learning has also been applied to manipulator path planning; the authors in [32] proposed Deep Deterministic Policy Gradient (DDPG) and Twin Synchro-Control (TSC) algorithms to achieve the fast-continuous path planning of a dual-manipulator system for multiple tasks. Moreover, those of [33] presented a single robot arm path planning algorithm using a Twin Delayed Deep Deterministic Policy Gradient (TD3) with Hindsight Experience Replay (HER) for a smoother path. With the aforementioned applications, we hope that AI technology can also make progress in the self-collision detection of a dual-manipulator system.

In this paper, we propose corresponding solutions to the above problems: (a) the GJK algorithm was introduced to solve the problem of insufficient accuracy of self-collision detection. (b) By introducing AI technology, a two-level self-collision detection algorithm is proposed, which improves the efficiency of detection. To improve the accuracy of collider modeling, the regular shaped bounding box was not applied in our research. We chose appropriate convex point sets on the surface of the manipulators, and the point sets were divided into multiple convex polyhedrons as the colliders of self-collision detection. It is worth noting that this paper represents the first use of deep learning for the self-collision detection of a dual-manipulator system under geometric simulation. A deep neural network, DLNet, was trained to improve the detection efficiency of the GJK algorithm. First, we generated all working states for the dual-manipulator system in its workspace and detected self-collision with the GJK algorithm in these states. Therefore, we obtained the self-collision state dataset of the workspace. Then, we used the dataset for training DLNet, which can be applied directly to judge self-collision risk. Finally, the trained DLNet and GJK algorithm were combined into a two-level self-collision detection algorithm, the DLGJK algorithm, to solve the real-time self-collision detection problem in a dual-manipulator system with fast-continuous and high-precision properties. The DLGJK algorithm takes the joint motor configuration of each manipulator as input and has autonomous judgment capability. DLNet firstly outputs a Boolean result for self-collision risk. For the working state with a self-collision risk, the DLGJK algorithm enters the second level of detection, which comprises calling the GJK algorithm to perform self-collision detection. The experimental results show that compared to that with the global use of the GJK algorithm, the DLGJK algorithm significantly increases the detection efficiency in both single detection and working-path detection. In particular, the time expectation for single detection of the workspace was reduced by 97.7%. At the same time, it was proven in experiments that the DLGJK algorithm can be applied to a multi-manipulator system, which can be split into dual-manipulator systems.

The rest of this paper is organized as follow: in Section 2, we introduce the multi-manipulator system used in this paper and introduce its kinematic modeling and the generation of colliders with high accuracy. In Section 3, we introduce the GJK algorithm and the process of collision detection mediated by the GJK algorithm. In Section 4, we introduce the process of the DLGJK algorithm and generate the training dataset. Then, we introduce the structure of DLNet and train it. In Section 5, we provide the experimental results, and the conclusion is given in Section 6.

## 2. Multi-Manipulator System and Collider Modeling

### 2.1. Kinematic Modeling of Multi-Manipulator System

**Figure 1:**
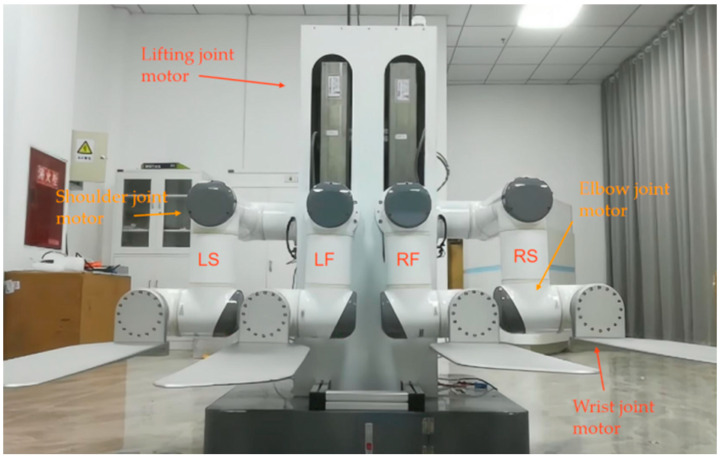
Front view of four-manipulator system.

As shown in Figure 1, the research in this paper was based on a mobile handling robot with four-manipulators attached. The four manipulators were named LS, LF, RF, and RS, which represents the left side arm, left front arm, right front arm, and right side arm, respectively. The load capacity of each manipulator could reach 50 kg. The robot uses SolidWorks for structural design and is manufactured in strict accordance with the design parameters.

There are two modes for the robot control program to control the manipulators. The first is that the manipulators move according to the specified path, and each working state in the path performs self-collision detection during path planning. The second is the real-time control mode, in which the control program controls the free movement of the manipulators in real time at the frequency of 50 times/s. In this mode, it is necessary to perform self-collision detection based on the working state in the command before each command is sent. Only the command without self-collision will be sent to the robot. With this demand, geometric simulation-based self-collision detection is more suitable for this paper.

In real-time motion planning, there is a risk of collision between two adjacent manipulators. The four-manipulator system can be divided to three dual-manipulator systems, LF-RF, RF-RS, and LF-LS. We illustrate the self-collision detection algorithm with the LF-RF dual-manipulator system.

**Figure 2:**
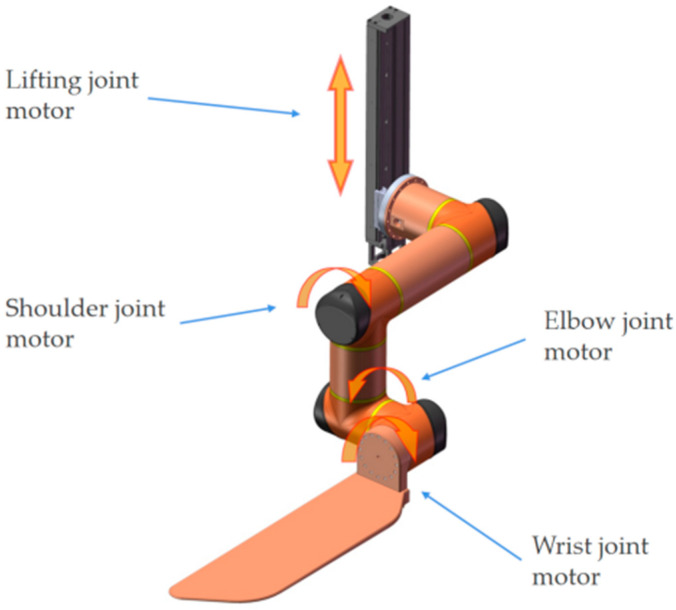
Single manipulator joint motor position.

As shown in Figure 2, each manipulator consists of four joints: the lifting joint (prismatic joint), shoulder joint, elbow joint, and wrist joint, and the wrist joint is attached to a replaceable end-effector. The configuration of the joint motor is shown in Table 1. The self-collision in the dual-manipulator system is influenced by the lift joint and shoulder joint: LF_1_ represents the LF lifting joint motor value, LF_2_ represents the LF shoulder joint motor value, RF_1_ represents the RF lifting joint motor value, and RF_2_ represents the RF shoulder joint motor value.

**Figure 3:**
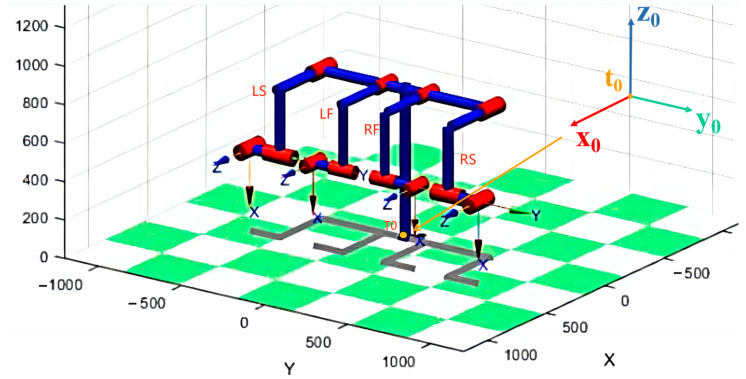
D-H model of four-manipulator system and base coordinate system **T_0_**.

The D-H parameters in mechanical engineering are the four parameters associated with a particular convention, for attaching reference frames to the links of a spatial kinematic chain or robot manipulator. In this paper, as shown in Figure 3, we used the D-H method in MATLAB to model the four-manipulator system. The base coordinate system is named **T_0_**. The origin of **T_0_** is the center of the robot chassis. We took the vertical direction pointing upwards as the positive direction of the **T_0_-Z** axis and the robot’s moving forward direction as the positive direction of the **T_0_-X** axis.

**Figure 4:**
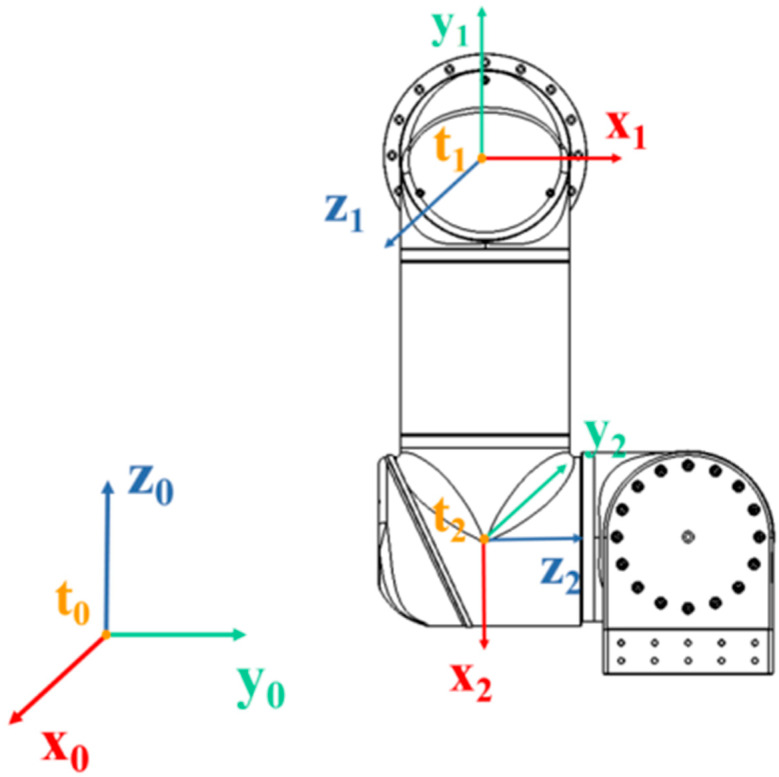
Front view of **T_0_** and RF joint coordinate system RF-T_1_, RF-T_2_.

It should be noted that the four-manipulator system in this paper has only one base coordinate system, **T_0_**, while each manipulator has its own joint coordinate system, **T_i_** (i > 0). We took RF as an example, and the base coordinate system **T_0_** and RF joint coordinate system **RF-T_1_**, **RF-T_2_** are shown in Figure 4. The D-H parameters of RF and LF are shown in Table 2.

Based on the D-H parameters, the kinematic model of each manipulator was established, and the pose transformation matrix between two links *i* and *i* + 1 was obtained as follows:

(1) Tii−1=[cosθi−sinθicosαisinθisinαiaicosθisinθicosθicosαi−cosθisinαiaisinθi0sinαicosαidi0001]

To calculate the transformation relationship between **T_i_** and **T_0_**, we established the pose transformation matrix of the *i*-th link in space as follows:

(2) Ti0=T10T21T32⋯Tii−1

### 2.2. Collider Construction of Manipulators

Conventional geometric simulation-based methods use regular-shaped bounding boxes, such as spheres, cylinders, and cubes as colliders. The modeling of these colliders is simple, and the algorithm for distance calculation is relatively simple. For example, the distance between spheres can be converted to calculate the distance between the centers of spheres, and the distance between cylinders can be converted to calculate the distance between axes [34]. However, this modeling method of the colliders will affect the modeling accuracy at the irregular outer surface, thereby affecting the self-collision detection accuracy at these positions. If the distances between colliders are large and the loss of accuracy at these positions is acceptable, these methods can be used for collision detection.

Different from other studies, there were irregular outer surfaces at the joints of the manipulators used in this paper. These irregular surfaces were only a few millimeters away from the other manipulator in many working states. Therefore, these positions were the focus areas of this paper. Our task requirements exceeded the detection accuracy of conventional geometric simulation-based methods, resulting in undetected self-collisions that have occurred or false self-collision warnings. These conventional methods are not suitable for our manipulators, and we needed to study a method with higher detection accuracy.

**Figure 5:**
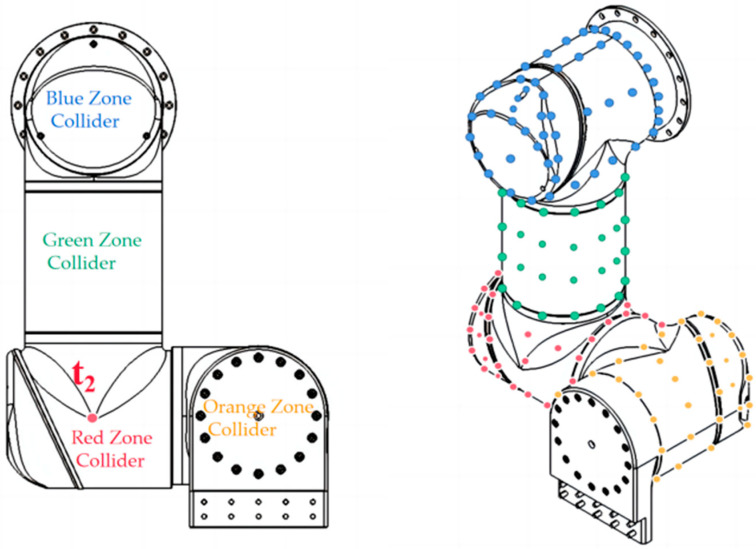
The point set of RF colliders.

To improve the detection accuracy, we selected a certain number of points on the surface of each manipulator to envelop them. The selected points of each manipulator were accurately measured, calculated using the SolidWorks (version 2021) modeling software and confirmed on the actual robot. After the high-precision collider modeling of the manipulators, the distance calculation method based on the regular-shape collider cannot be used, and thus, we introduced the GJK algorithm. Since the GJK algorithm can only detect the collision relationship between convex shapes, as shown in Figure 5, each manipulator was divided into multiple convex colliders. All colliders of manipulators were established in their own **T_2_** coordinate system.

In order to detect the spatial position relationship between the colliders of each manipulator, these colliders needed to be converted from **T_2_** to **T_0_**. Therefore, the pose transformation matrix of each manipulator needed to be calculated. Taking RF as an example, from Equation (1) we can obtain:

(3) T10RF=[0010100125010d1RF+7800001]

(4) T21RF=[cos(θ2RF−90)0−sin(θ2RF−90)346cos(θ2RF−90)sin(θ2RF−90)00346sin(θ2RF−90)0−1cos(θ2RF−90)3510001]

Then, from Equation (2) we can obtain the transformation matrix between the **RF-T_2_** and **T_0_** as follows:

(5) T20RF=T10RFT21RF=[0−1sin(θ2RF)351sin(θ2RF)0cos (θ2RF)346sin(θ2RF)+125−cos (θ2RF)00−346cos (θ2RF)+d1RF+7800001]

According to the robot forward kinematics, with the manipulator joint motors angle data, we can calculate the matrix T20 in real-time:

(6) pos0=T20⋅pos2 

According to Equation (6), we can convert the coordinates of the colliders from **T_2_** to the uniform base frame **T_0_**, where $pos_{0}$ represents the generated point set of colliders in **T_0_** and $pos_{2}$ represents the generated point set of colliders in **T_2_**. Hence, during the movement of the system, we can obtain the point sets representing corresponding colliders in **T_0_** in real-time.

## 3. GJK Algorithm for Dual-Manipulator Self-Collision Detection

### 3.1. Introduction of GJK Algorithm

#### 3.1.1. Minkowski Difference

Before introducing the GJK algorithm, we first introduced the Minkowski difference. Assuming A and B are two convex polyhedrons in Cartesian space, a is a vector in A and b is a vector in B. The Minkowski difference between A and B is defined as:

(7) A−B={a−b|a∈A,b∈B}

We named the convex polyhedron formed by A−B as C, C=A−B. The distance between A and B can be expressed as follows:

(8) d(A,B)=min{‖ x - y ‖ : x∈A,y∈B}

assuming that v(C) represents the point nearest to the origin in C and satisfies the following equation:

(9) ‖v(C)‖=min{‖x‖:x∈C} 

According to Equations (8) and (9), we obtained:

(10) d(A,B)=v(C)

Which proves that calculating the minimum distance between A and B can be translated into determining whether C contains the origin.

In other words, if there is a collision between convex polyhedron A and B, the convex polyhedron C (C=A−B) must contain the origin. This is a very important property of the Minkowski difference in convex polyhedron collision detection.

#### 3.1.2. Basic Principle of GJK Algorithm

Before describing the basic principle of the GJK algorithm, we needed to understand two definitions.

**Definition** **1:** *Point P belongs to the convex polyhedron*C*. For a given direction vector d, if point P satisfies equation:*

(11) d⋅P=max{d⋅V|V∈C}

*then point P is called the support point of*C*in direction d. The function to find the support point is called the support function, written as $S_{\left(C\right)}$, the finding direction is written as*$V_{dir}$.

**Definition** **2:** *For convex polyhedron* C *, a simplex is a convex tetrahedron formed by any four vertices in* C *. If the selected vertices are different, the simplex formed is also different. Selected vertex* q *is constructed by* $S_{\left(C\right)}$ *along different* $V_{dir}$ *and satisfies the equation:*

(12) $$q=S_{\left(C\right)}(A,V_{dir})-S_{\left(C\right)}(B,V_{dir})\;$$

The GJK algorithm uses the Minkowski difference property described in Section 3.1.1 to compute the minimum distance between two convex polyhedrons. For convex polyhedron A and B, the GJK algorithm iteratively searches the point with the closest distance to the origin in C (C=A−B). The GJK algorithm generates a simplex in every iteration process, and the simplex generated at the *k*-th iteration process is denoted as $W_{k}$. $v_{k}$ is the point nearest to the origin in $W_{k}$ and can be calculated by choosing the Johnson operator [21] or the improved operator [28,35,36] depending on the situation. If $v_{k}$ is the origin, then $W_{k}$ contains the origin, which means that C contains the origin, and thus, a collision occurs between A and B. If $v_{k}$ is not the origin, then the algorithm updates $V_{dir}$ according to the rule and obtains the new vertex $q_{k+1}$, replacing a vertex in $W_{k}$ with $q_{k+1}$ to get $W_{k+1}$ and continue to determine whether $W_{k+1}$ contains the origin.

The GJK algorithm terminates the loop in two cases:

(a) $v_{k}$ is the origin, A and B collide, and the GJK algorithm is exited;
(b) The dot product of $q_{k+1}$ and $V_{dir}$ is less than zero ($\mathrm{dot}(V_{q_{k+1}O},V_{dir})<0$), at this time, the simplex containing the origin cannot be found in C, no collision occurs between A and B, and the GJK algorithm is exited.

### 3.2. GJK Self-Collision Detection for Dual-Manipulator System

**Figure 6:**
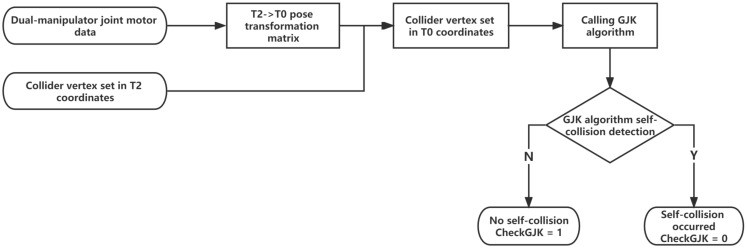
Flowchart of GJK algorithm self-collision detection.

As shown in Figure 6, by inputting the real-time joint motor configuration (height data and angle data) of the dual-manipulator system, the transformation matrix between **T_0_** and **T_2_** are obtained. After converting all colliders to a unified coordinate system, the GJK algorithm determines the real-time self-collision detection results of the colliders. The collision mark is recorded as CheckGJK, which is equal to 1 when a self-collision occurs.

## 4. DLGJK Algorithm

### 4.1. Structure of DLGJK Algorithm

**Figure 7:**
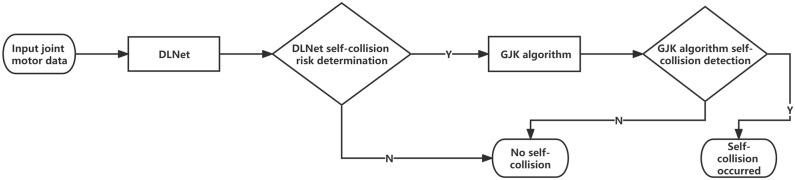
Flowchart of DLGJK algorithm self-collision detection.

Figure 7 shows the flowchart of the DLGJK algorithm. The DLGJK algorithm consists of DLNet and the GJK algorithm, and the input of the DLGJK algorithm is the real-time joint motors configuration of the dual-manipulator system. First, the DLGJK algorithm uses DLNet to make a judgment, and if there is no self-collision risk, the DLGJK algorithm is directly quit; if there is a risk of self-collision, the DLGJK algorithm calls the GJK algorithm to perform self-collision detection, and the GJK algorithm will detect whether self-collision occurs in the current working state.

The overall workflow of collision detection consists of two layers; the first layer is DLNet for risk checking, the second layer is GJK for risky situations, which are determined as such from first layer DLNet.

The segment judgment process of DLNet is closer to human thinking. When we judge whether there is a collision between manipulators, we present the judgment that there is no self-collision risk for manipulators with a long distance. As the distance between the manipulators gets closer and closer, we will present the judgment that there is a risk of self-collision and that self-collision detection is needed. After DLNet learns the relationship between workspace self-collision states and joint motors data, the motors data can replace the distance as the judgment basis of DLNet, so that DLNet can imitate our thinking logic for self-collision risk judgment.

**Figure 8:**
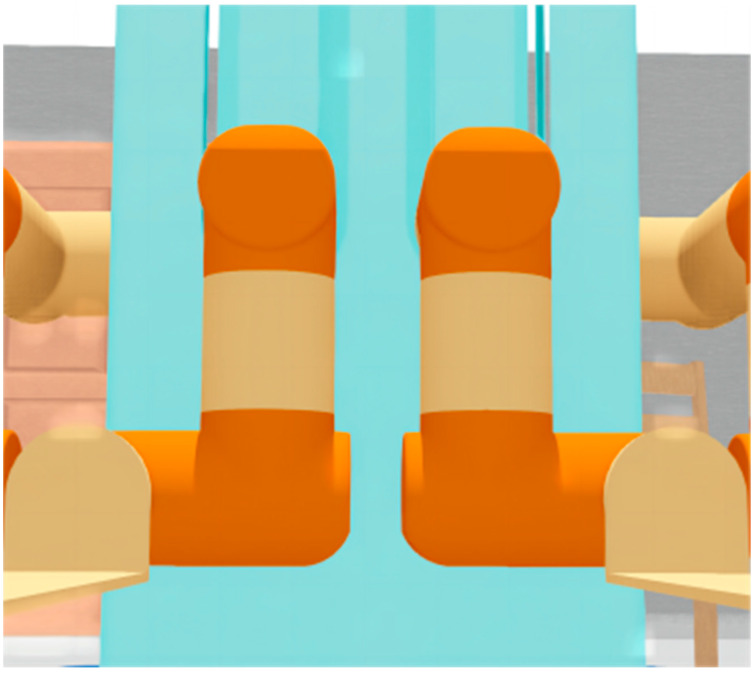
Dual-manipulator LF-RF without risk of self-collision.

Ideally, the working state of the dual-manipulator system and the self-collision detection result of the DLGJK algorithm should contain the following three cases:

As shown in Figure 8, the system has no self-collision risk and no self-collision occurs: the DLNet judges that there is no self-collision and the DLGJK algorithm returns the information that no self-collision is detected.

**Figure 9:**
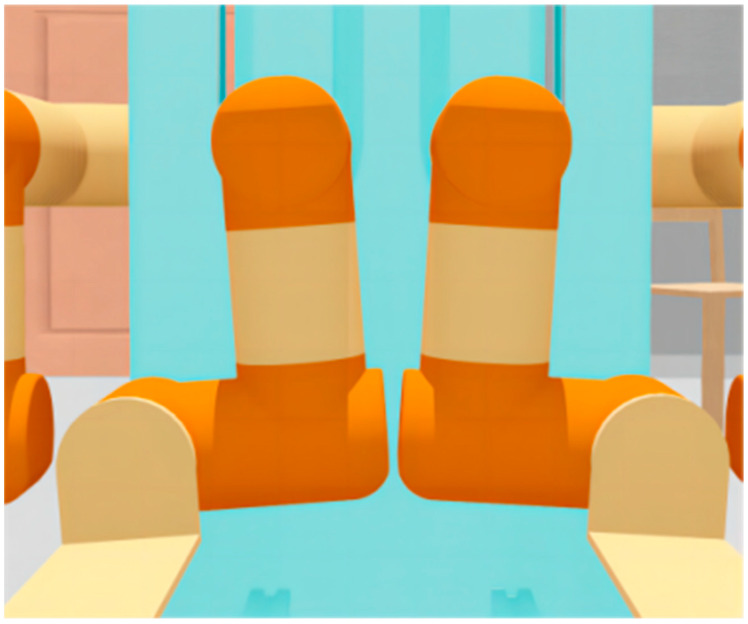
Dual-manipulator LF-RF with risk of self-collision.

As shown in Figure 9, the system has a self-collision risk, but no self-collision occurs: the DLNet judges that there is a self-collision risk and the GJK algorithm does not detect a self-collision, finally the DLGJK algorithm returns the information that no self-collision is detected.

**Figure 10:**
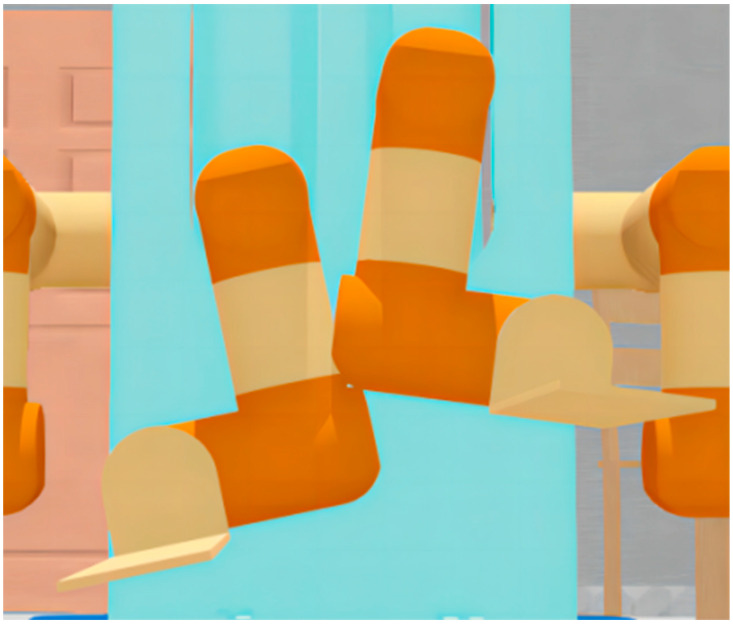
Dual-manipulator LF-RF with self-collision.

As shown in Figure 10, a self-collision occurs in the system: the DLNet judges that there is a risk of self-collision, the GJK algorithm detects a self-collision, and finally the DLGJK algorithm returns the information that a self-collision occurs.

Therefore, the DLNet must be accurate in judging the no-self-collision working state. That is, if DLNet judges that there is no risk of self-collision, the dual-manipulator system must be in a no self-collision state; if the DLNet judges that there is a risk of self-collision, the system may have a self-collision. Then, the DLGJK algorithm must call the GJK algorithm for self-collision detection and returns the final result. The implementation logic will be introduced in the Section 4.3.

### 4.2. Structure and Training of DLNet

#### 4.2.1. Gathering DLNet Training Data

In order to obtain the dataset required for training the DLNet, it is necessary to generate the workspace data of the dual-manipulator system. As described in the previous section, the self-collision of the system studied in this paper is mainly affected by the motor motions of the lifting joint motors (LF_1_, RF_1_) and shoulder joint motors (LF_2_, RF_2_). According to the value ranges and step amounts of LF_1_, LF_2_, RF_1_, and RF_2_, the dual-manipulator system workspace dataset is generated exhaustively, and the GJK algorithm is called to perform self-collision detection on all data. Finally, the self-collision state dataset of the workspace in the format [LF_1_, LF_2_, RF_1_, RF_2_, CheckGJK] is obtained, written as the Space-Col dataset.

**Figure 11:**
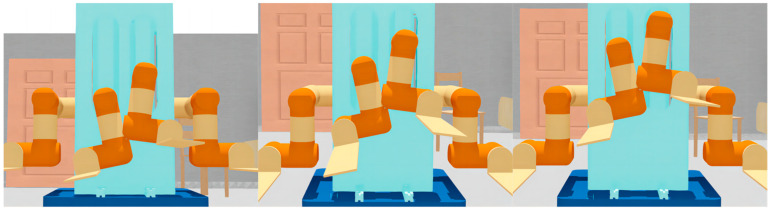
Dual-manipulator LF-RF lift the same height at the same time.

The study of the Space-Col dataset shows that for the dual-manipulator system used in this paper, when LF_2_ and RF_2_ are constant and the height difference between LF_1_ and RF_1_ is unchanged, the values of LF_1_ and RF_1_ have no effect on the self-collision state. That means only three variables: the height difference between LF_1_ and RF_1_, the LF_2_, and the RF_2_ can represent the relative states between two manipulators. As shown in Figure 11, the above conclusion means that, under the condition that the angle of the shoulder joint motors is unchanged, two manipulators lifting or falling the same height at the same time will not change the self-collision state.

The height difference between LF_1_ and RF_1_ is denoted as the Hvalue. We used the Hvalue to replace LF_1_ and RF_1_ in the Space-Col dataset. After the data were de-duplicated, the training dataset in the format of [Hvalue, LF_2_, RF_2_, CheckGJK] was obtained and written as the DL-Train dataset.

#### 4.2.2. Structure and Parameters of DLNet

The DL-Train dataset was used to train the DLNet. According to the characteristics of the dataset, we used a five-layer fully connected neural network to construct DLNet. The x-input of DLNet is the Hvalue, LF_2_, and RF_2_, and the y-input is CheckGJK. The output of the network is the probability of self-collision of the dual-manipulator system in the respective working state, denoted as OutDL.

**Figure 12:**
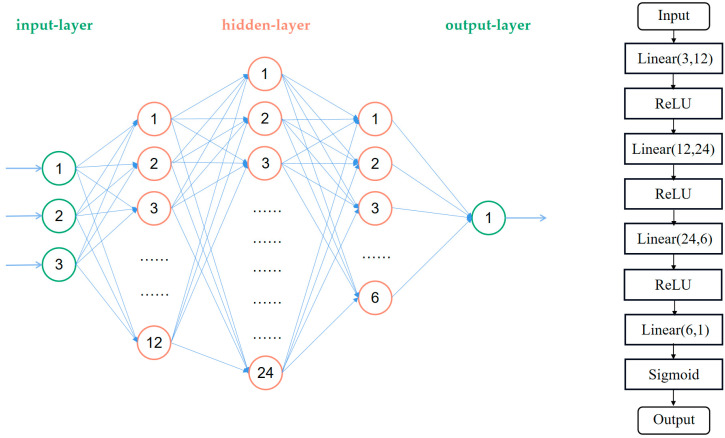
Topology and model structure of DLNet.

The DLNet includes the input-layer, hidden-layer, and output-layer. The number of neurons in the input-layer is set to three (x-input) and the number of neurons in the output-layer is set to one (OutDL). The trial-and-error method was used to determine the number of hidden-layers and the number of neurons in the hidden-layers. The final number of hidden-layers was determined to be three, and the numbers of neurons were 12, 24, and 6, respectively. As shown in Figure 12, the final topology of DLNet was determined to be 3:12:24:6:1.

For the selection of the activation function, the ReLU function that enables faster network training was selected as the activation function of hidden-layers, while the output of the output-layer is essentially a binary problem; therefore, the Sigmoid function, which is more suitable for the binary problem, was selected as the activation function of the output-layer. Since the DL-Train dataset features are clearly distributed, after experimental verification, the Stochastic Gradient Descent (SGD) was selected as the optimization method and the BinaryCrossEntropyLoss (BCELoss) function was selected as the loss function.

**Figure 13:**
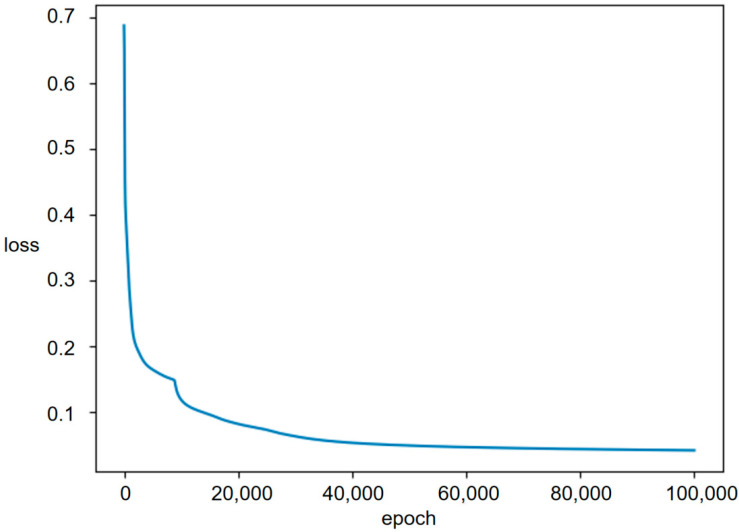
Change in the loss value during DLNet training.

It is worth noting that since the DL-Train dataset actually contains all the working states of the dual-manipulator system used in this paper, it is unnecessary to consider the overfitting problem. As long as the DLNet can learn the DL-Train dataset well, it can judge all working states of the dual-manipulator system. Model training proceeds until the loss value converges, and the loss changes in the training process are shown in Figure 13. In this paper, accuracy was not an important indicator for evaluating the DLNet training results. We will select a threshold in the following part and process the network output to achieve 100% accuracy in judging no self-collision working states.

### 4.3. Judgment Logic of DLNet for Self-Collision

As described in Section 4.1, the DLNet must be accurate in judging the no-self-collision working state. The output value (OutDL) of the DLNet is the predicted value of self-collision in the current working state. The value of OutDL has a range of [0, 1], where OutDL = 1 means that no self-collision occurs and OutDL = 0 means that self-collision occurs. The closer the OutDL is to 0, the higher the probability of self-collision occurring. Associating the model x-input with the OutDL, we obtained the DLNet output dataset in the format [Hvalue, LF2, RF2, OutDL], written as the DL-Out dataset.

As the self-collision judgment basis of the DLNet, we need to select a critical threshold (K) between 0 and 1. If 0 < OutDL ≤ K, the DLNet judges that there is a self-collision risk; if K < OutDL ≤ 1, the DLNet judges that there is no self-collision risk. The selection of K with this logic should satisfy the following requirements:

0 ≤ OutDL ≤ K, the DLNet judges that there is a risk of self-collision, and at this time, the dual-manipulator system should be in a state with a risk of self-collision or a self-collision has occurred, and the final detection result of the DLGJK algorithm needs to be given by the GJK algorithm.

K < OutDL ≤ 1, the DLNet judges that there is no risk of self-collision, and at this time, the dual-manipulator system should be in a state without self-collision risk, and the final detection result of the DLGJK algorithm is directly given by the DLNet.

Thus, the key point is: for a selected K, for all data in the DL-Out dataset that satisfy K < OutDL ≤ 1, the detection result given by the GJK algorithm (CheckGJK) should be equal to 1. Therefore, the verification method for whether this K satisfies the requirements is as follows: determine all data in the DL-Out dataset that meet K < OutDL ≤ 1, map these data to the DL-Train dataset, and verify whether all corresponding CheckGJK values are equal to 1. If the CheckGJK values of all data are equal to 1, this K satisfies the requirements.

**Figure 14:**
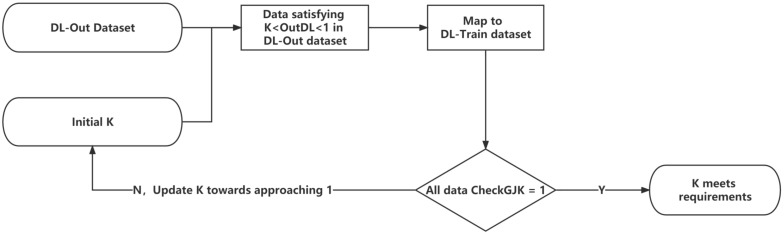
Flowchart for finding the K-value that satisfies the requirements.

For the DLNet, the range of K to satisfy the requirements should be an interval belonging to (0, 1). As shown in Figure 14, the search process for K can gradually approach 1 through dichotomy and finally find the K that satisfies the requirements.

**Figure 15:**
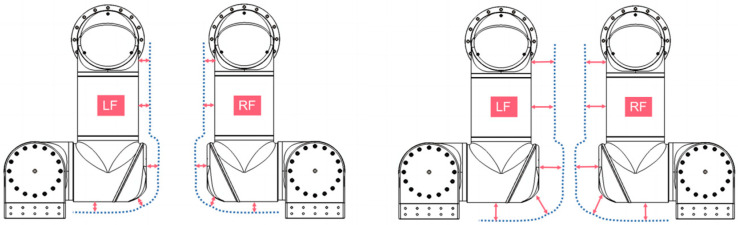
The detection distance increases with an increase in K.

Assuming that the minimum satisfying K is K_min_, as shown in Figure 15, the selected K gradually approaches from K_min_ to 1, and the DLNet is more and more cautious in judging self-collision. At the same time, the self-collision judgement distance of colliders will be larger and larger, and the GJK algorithm will be called more often for self-collision detection. We can adjust the judgement distance of DLNet for self-collision by adjusting K.

The pseudo-code of the DLGJK algorithm for self-collision judgment of the LF-RF dual-manipulator system (Algorithm 1) is as follows:
**Algorithm 1** DLGJK self-collision detection of the LF-RF dual-manipulator system1.  Input: motor data, containing motor data of 8 joints of the system. 2.  Extract [LF_1_, LF_2_, RF_1_, RF_2_]. 3.  Hvalue ← LF_1_-RF_1_. 4.  Call the DLNet, input [Hvalue, LF_2_, RF_2_] If OutDL > K: Return no self-collision occurs in the system, exit DLGJK algorithm; If OutDL ≤ K: Continue to execute the next step. 5.  Call GJK algorithm, input [LF_1_, LF_2_, RF_1_, RF_2_] If GJK algorithm detects no self-collision: Return no self-collision occurs in the system, exit DLGJK algorithm; If GJK algorithm detects the occurrence of self-collision: Return self-collision occurs in the system, exit DLGJK algorithm.

The four-manipulator system used in this paper can be regarded as three dual-manipulator systems and can perform self-collision detection simultaneously in the control system. Since the initial distance and relative position between each pair of manipulators are different, the DLNet and K (the K below refers to K_min_) should be retrained for different dual-manipulator systems. The pseudo-code of the DLGJK algorithm for self-collision detection of the four- manipulator system (Algorithm 2) is as follows:
**Algorithm 2** DLGJK self-collision detection of the four-manipulator system1.  Input: motor data, containing motor data of 16 joints of the system. 2.  Extract [LF_1_, LF_2_, RF_1_, RF_2_], [RS_1_, RS_2_, RF_1_, RF_2_], [LS_1_, LS_2_, LF_1_, LF_2_]. 3.  Apply DLGJK algorithm simultaneously for three groups of dual-manipulator systems:  DLGJK algorithm detection for LF-RF, input [LF_1_, LF_2_, RF_1_, RF_2_]    If self-collision is detected:      Return LF-RF occurs self-collision, exit DLGJK algorithm;    If no self-collision is detected:      Return LF-RF no self-collision;  DLGJK algorithm detection for RS-RF, input [RS_1_, RS_2_, RF_1_, RF_2_]    If self-collision is detected:      Return RS-RF occurs self-collision, exit DLGJK algorithm;    If no self-collision is detected:      Return RS-RF no self-collision;  DLGJK algorithm detection for LS-LF, input [LS_1_, LS_2_, LF_1_, LF_2_]    If self-collision is detected:      Return LS-LF occurs self-collision, exit DLGJK algorithm;    If no collision is detected:      Return LS-LF no self-collision; 4.  If no self-collision occurs in the three groups of dual-manipulator systems, then no self-collision occurs in the four-manipulator system, exit DLGJK algorithm.

## 5. Experiment and Discussion

### 5.1. Experimental Platform and Simulation Environment

The research in this paper was based on a mobile handling robot with four manipulators. The simulation system environment is Windows 10 × 64, Intel i5-11600KF 3.90 GHz, DDR4 64.0 GB, NVIDIA GeForce RTX 3070 Ti, and 1T SSD. The deep-learning environment is based on the python3.9 pytorch framework, version 1.11.0. The robot control program was written on QT Creator platform, version 5.15.2, and the programming language is C++. The simulation software is Webots, version 2021b.

**Figure 16:**
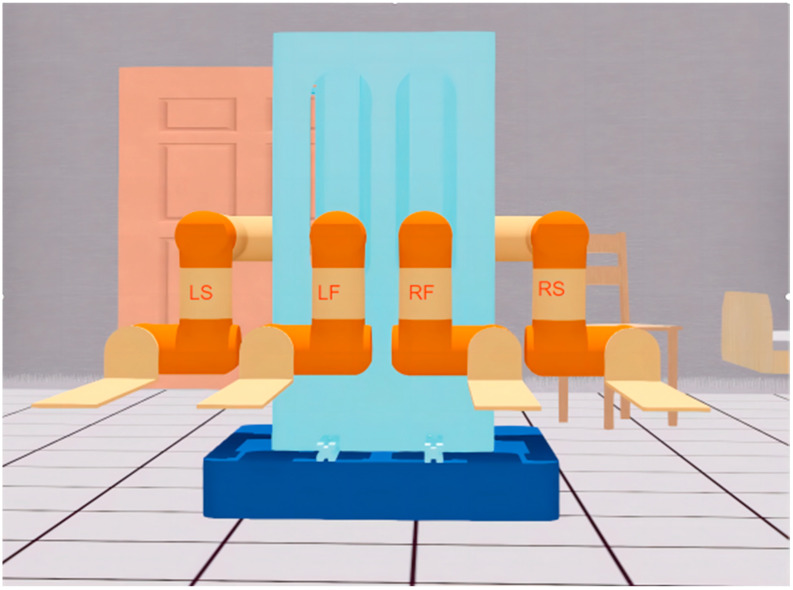
Webots model of four-manipulator handling robot.

The simulation environment should be as close as possible to the real physical environment, so that the simulation manipulator can reflect the situation of the real manipulator in real time and ensure that the algorithms and data in the simulation environment can be used in the real environment. We directly imported the output model of SolidWorks into Webots to ensure a high degree of unity among the SolidWorks model, Webots model, and real robot. After our measurement and test, there was no visible error between the physical environment robot and the simulation environment robot. Figure 16 shows the Webots simulation model.

### 5.2. Single Detection Time of DLGJK Algorithm

#### 5.2.1. Single Detection Time of DLNet and GJK Algorithm

In this section, we calculated the single-detection time by dividing the detection time of the dataset by the amount of data in the dataset. We took RF-LF as an example; since the DL-Train dataset had covered the entire workspace of RF-LF, we used the DLNet to detect the DL-Train dataset ten times. For a comparison, we used the GJK algorithm to detect the Space-Col dataset ten times as well, in order to control variables; data with the same amount as the DL-Train dataset were randomly selected from the Space-Col dataset for the GJK algorithm. The results of ten detection times are shown in Table 3.

As shown in Table 4, the average single-detection time of DLNet (T_DL_) is 0.12 μs, and the average single-detection time of the GJK algorithm (T_GJK_) is 1129 μs, and the judgment speed of DLNet for self-collision is much faster than the detection speed of the GJK algorithm, which is one of the reasons why the DLGJK algorithm can improve detection efficiency.

#### 5.2.2. Theoretical Single-Detection Time of DLGJK Algorithm

According to the working logic of the DLGJK algorithm, the single-detection time of DLGJK algorithm should be discussed in different situations: for the working states without a self-collision risk, the single-detection time of the DLGJK algorithm is denoted as T_1_, T_1_ = T_DL_ = 0.12 μs; for the working states with a self-collision risk or self-collision occurrence, the single-detection time of the DLGJK algorithm is denoted as T_2_, T_2_ = T_DL_ + T_GJK_ = 1129.12 μs, with the results recorded in Table 5. We could observe that the DLGJK algorithm takes much less time than the GJK algorithm in a single detection for the state without a self-collision risk and does not increase the detection time for the state with a self-collision risk.

#### 5.2.3. Actual Single-Detection Time of the DLGJK Algorithm

For the workspace of the RF-LF dual-manipulator system, the single-detection time of the DLGJK algorithm should be calculated as the time mathematical expectation of its single detection, denoted as:

(13) $$Ε(T_{\mathrm{DLGJK}})=T_{1}(1-P)+T_{2}P$$

P is the probability of the DLGJK algorithm calling GJK algorithm in a single detection. We used the DLGJK algorithm and DL-Train dataset to detect the self-collision states of the RF-LF workspace. In the DLGJK algorithm, the first-level frequency is the frequency of calling DLNet, which is called globally during algorithm execution; the second-level frequency is the frequency of calling the GJK algorithm, which is called according to the judgment result of DLNet.

Compared to the global use of the GJK algorithm, the probability of the DLGJK algorithm calling the GJK algorithm and the time expectation of a single self-collision detection are shown in Table 6.

We can observe that for the RF-LF system, compared to that with the global use of the GJK algorithm, the single self-collision detection time when using the DLGJK algorithm is reduced by 97.7%, and the number of times calling the GJK algorithm (DLGJK second-level frequency) is effectively reduced, which reduces the system overhead.

For the four-manipulator system, we used the same method to calculate the single-detection time expectation for LF-LS and RF-RS, and the results are shown in Table 7 and Table 8.

**Figure 17:**
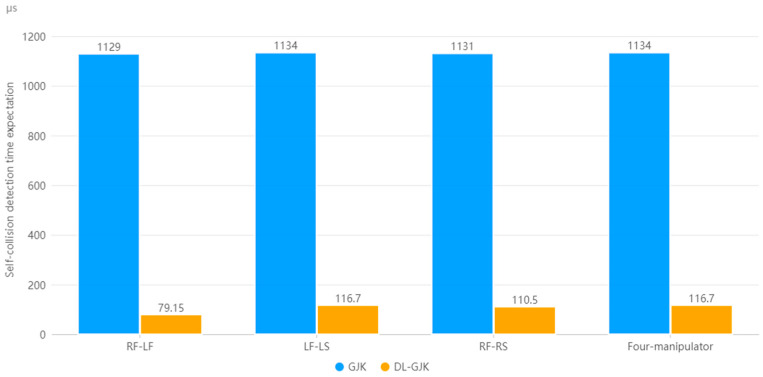
Single-detection time expectation of system workspace.

Since the collision detection of each group of dual-manipulator systems is calculated in parallel in the robot control system, as 39.81 μs, the maximum values of E_(TDLGJK)_, E_(TDLGJK)(RF-RS)_, and E_(TDLGJK)(LF-LS)_ are taken as the time expectation of the DLGJK algorithm single detection for the four-manipulator system. As shown in Figure 17, compared to that when using the GJK algorithm globally, using the DLGJK algorithm can significantly reduce the single-detection time expectation, improve the detection efficiency, and effectively reduce the number of times calling the GJK algorithm.

For the real-time control system, the single-detection time of the DLGJK algorithm meets the requirement, and the detection speed far exceeds the standard for most of the working states.

### 5.3. DLGJK Algorithm Self-Collision Detection for Working Path

Self-collision detection is an important process of path planning for multiple manipulators. In this part, we use the DLGJK algorithm to detect the working path of the dual-manipulator system and the four-manipulator system. The global GJK algorithm can also be used for comparison.

**Figure 18:**
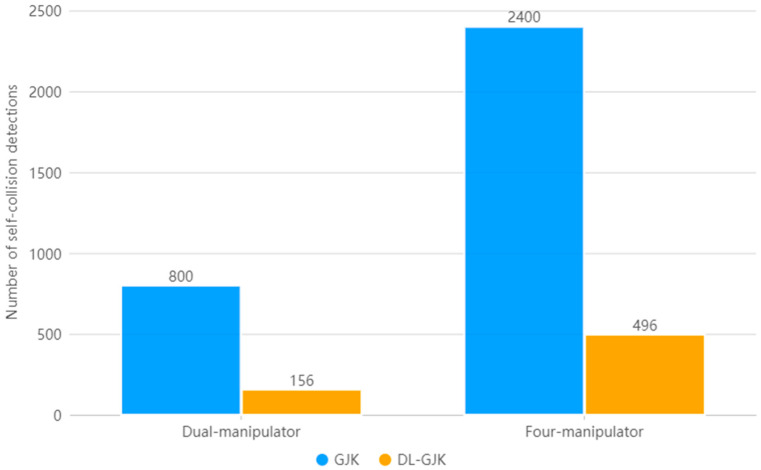
Number of detections for manipulator working path.

For the dual-manipulator system, a working path consisting of 800 motion-state sequences in the actual task of RF-LF is used. For the four-manipulator system, we also used a working path consisting of 800 motion-state sequences, which is a total of 2400 motion-state sequences for the three groups of dual-manipulator systems. The experimental results are shown in Figure 18.

During the movement of the RF-LF, the GJK algorithm is called 800 times when the GJK algorithm is used globally, while it is called 156 times when the DLGJK algorithm is used. When using the DLGJK algorithm, the number of self-collision detections is reduced by 80.5%.

During the movement of the whole system, the GJK algorithm is called 2400 times when the GJK algorithm is used globally and 496 times when the DLGJK algorithm is used. In the case of using the DLGJK algorithm, the number of self-collision detections is reduced by 79.4%.

The experimental results show that for the system used in this paper, since most of the working states in the working path have no self-collision risk, compared to that when using the GJK algorithm globally, the DLGJK algorithm improves detection efficiency by saving the number of detections with no self-collision risk. At the same time, it can effectively reduce the time spent on self-collision detection and reduce the system overhead.

## 6. Conclusions

To solve the problem of real-time self-collision detection with high-precision in a multi-manipulator control system, we propose a two-level self-collision detection algorithm based on the GJK algorithm and deep learning, the DLGJK algorithm. The proposed algorithm has made great progress in the accuracy and efficiency of self-collision detection. When applying the DLGJK algorithm for self-collision detection, the DLNet is firstly used to independently judge whether there is self-collision risk in the current working state of the system. For the working state without a self-collision risk, the GJK algorithm is not called; for the working state with a self-collision risk, the DLGJK algorithm enters the second level of detection such that the GJK algorithm is called to perform self-collision detection.

For the dual-manipulator system, the experimental results show that the DLGJK algorithm takes much less single-detection time than the GJK algorithm for the working state without self-collision and does not increase the detection time for the working state with a self-collision risk. For the system workspace, compared to that with the global use of the GJK algorithm, DLGJK algorithm can reduce the single-detection time expectation by 97.7%. For the working path, the DLGJK algorithm effectively reduces the number of self-collision detections, which improves the detection efficiency and reduces the system overhead in self-collision detection.

The proposed approach also has good scalability for multiple-manipulator systems that can be divided into dual-manipulator systems, and we used a four-manipulator system to verify this.

## Figures and Tables

**Table 1:** Motor configuration of single manipulator.

Motor Position	Operating Range	Motor Stepping Amount
Lifting joint motor	0~500 mm	10 mm
Shoulder joint motor	−90°~90°	0.5°
Elbow joint motor	−90°~90°	0.5°
Wrist joint motor	−90°~90°	0.5°

**Table 2:** D-H parameters of RF and LF.

Manipulator	Link	*θ*_i_/(°)	*d*_i_/mm	*a*_i_/mm	*α*_i_/(°)
RF	*l*_1_^RF^	90	*d*_1_ ^RF^ + 780	125	90
	*l*_2_^RF^	*θ*_2_^RF^ − 90	351	346	−90
	*l*_3_^RF^	*θ*_3_^RF^	182	0	90
	*l*_4_^RF^	*θ*_4_^RF^	0	0	0
LF	*l*_1_^LF^	90	*d*_1_ ^LF^ + 780	−125	90
	*l*_2_^LF^	*θ*_2_^LF^ + 90	351	346	−90
	*l*_3_^LF^	*θ*_3_^LF^	182	0	90
	*l*_4_^LF^	*θ*_4_^LF^	0	0	0

**Table 3:** Dataset collision detection time of DLNet and GJK (ten detection times).

Algorithm	1	2	3	4	5	6	7	8	9	10
GJK	15,020.7985 s	15,024.0230 s	15,011.7995 s	15,001.7229 s	15,003.6697 s	15,033.8485 s	15,046.9780 s	15,007.4476 s	15,021.8630 s	15,024.3327 s
DLNet	1.5952 s	1.5773 s	1.5603 s	1.6240 s	1.6471 s	1.5634 s	1.6002 s	1.6001 s	1.5970 s	1.6033 s

**Table 4:** Single self-collision detection time comparison of DLNet and GJK.

Algorithm	Dataset Data Amount	Average Dataset Detection Time	Average Single-Detection Time
GJK	13,292,742	15,019.6483 s	1129 μs
DLNet	13,292,742	1.5969 s	0.12 μs

**Table 5:** Single self-collision detection time of DLGJK.

Working Conditions	Time-Consuming Equation	Single-Detection Time
No self-collision risk	T_1_ = T_DL_	0.12 μs
With self-collision risk	T_2_ = T_DL_ + T_GJK_	1129.12 μs

**Table 6:** RF-LF workspace self-collision detection time expectation of Global GJK and DLGJK.

Algorithm	Number of Times Calling GJK	P	Single Detection-Time Expectation
Global GJK	13,292,742	100%	E(T_GJK_) = T_GJK_ = 1129 μs
DLGJK	306,463	2.3%	E(T_DLGJK_) = T_1_ × 97.7% + T_2_ × 2.3% = 26.09 μs

**Table 7:** LF-LS workspace self-collision detection time expectation of Global GJK and DLGJK.

Algorithm	Number of Times Calling GJK	Single-Detection Time	P	Single-Detection Time Expectation
Global GJK	13,292,742	T_GJK(LF-LS)_ = 1134 μs	100%	E(T_GJK_) = T_GJK(LF-LS)_ = 1134 μs
DLGJK	471,702	T_1(LF-LS)_ = T_DL(LF-LS)_ = 0.12 μs T_2(LF-LS)_ = T_DL(LF-LS)_ + T_GJK(LF-LS)_ = 1134.12 μs	3.5%	E(T_DLGJK_)_(LF-LS)_ = T_1(LF-LS)_ × 96.5% + T_2(LF-LS)_ × 3.5% = 39.81 μs

**Table 8:** RF-RS workspace self-collision detection time expectation of Global GJK and DLGJK.

Algorithm	Number of Times Calling GJK	Single Detection Time	P	Single-Detection Time Expectation
Global GJK	13,292,742	T_GJK(RF-RS)_ = 1131 μs	100%	E(T_GJK_) = T_GJK(RF-RS)_ = 1131 μs
DLGJK	450,981	T_1(RF-RS)_ = T_DL(RF-RS)_ = 0.12 μs T_2(RF-RS)_ = T_DL(RF-RS)_ + T_GJK(RF-RS)_ = 1131.12 μs	3.4%	E(T_DLGJK_)_(RF-RS)_ = T_1(RF-RS)_ × 96.6% + T_2(RF-RS)_ × 3.4% = 38.57 μs

## Data Availability

Not applicable.
